# A benchmark of text embedding models for semantic harmonization of Alzheimer's disease cohorts

**DOI:** 10.1016/j.tjpad.2025.100420

**Published:** 2025-12-01

**Authors:** Tim Adams, Yasamin Salimi, Mehmet Can Ay, Diego Valderrama, Marc Jacobs, Holger Fröhlich

**Affiliations:** a Department of Bioinformatics, Fraunhofer Institute for Algorithms and Scientific Computing (SCAI), Sankt, Augustin, 53757, Germany; b Bonn-Aachen International Center for IT, Rheinische Friedrich-Wilhelms-Universität Bonn, Bonn, Germany; c Institute for Digital Medicine, University Hospital Bonn, Bonn, Germany

**Keywords:** Harmonization, Alzheimer’s disease, Text-embeddings, Large language models

## Abstract

**Background:**

Harmonizing diverse healthcare datasets is a challenging task due to inconsistent naming conventions. Manual harmonization is time- and resource-intensive, limiting scalability for multi-cohort Alzheimer's Disease research. Large Language Models, or specifically text-embedding models, offer a promising solution, but their rapid development necessitates continuous, domain-specific benchmarking, especially since general established benchmarks lack clinical data harmonization use cases.

**Objectives:**

To evaluate how different text-embedding models perform for the harmonization of clinical variables.

**Design and setting:**

We created a novel benchmark to assess how well different Language Model embeddings can be used to harmonize cohort study metadata with an in-house Common Data Model that includes cohort-to-cohort mappings for a wide range of Alzheimer’s Disease cohorts. We evaluated five different state-of-the-art text embedding models for seven different data sets in the context of Alzheimer’s disease.

**Participants:**

No patient data were utilized for any of the analyses, as the evaluation was based on semantic harmonization of cohort metadata only.

**Measurements:**

Text descriptions of variables from different modalities were included for the analyses, namely clinical, lifestyle, demographics, and imaging.

**Results:**

Our benchmark results favored different models compared to general-purpose benchmarks. This suggests that models fine-tuned for generic tasks may not translate well to real-world data harmonization, particularly in Alzheimer’s disease. We propose guidelines to format metadata to facilitate manual or model-assisted data harmonization. We introduce an open-source library (https://github.com/SCAI-BIO/ADHTEB) and an interactive leaderboard (https://adhteb.scai.fraunhofer.de) to aid future model benchmarking.

**Conclusions:**

Our findings highlight the importance of domain-specific benchmarks for clinical data harmonization in the field of Alzheimer’s disease and motivate standards for naming conventions that may support semi-automated mapping applications in the future.

## 1 Introduction

As data availability in healthcare continues to expand, so does access to diverse, large-scale datasets collected across various institutions and populations. This growing wealth of information presents a unique opportunity to advance data-driven research and improve clinical decision-making. Published cohort studies, such as the Alzheimer's Disease Neuroimaging Initiative (ADNI) [1], have been shown to accelerate research [2] and enhance our understanding of disease progression by providing open access to high-quality, longitudinal data. However, individual cohorts are often biased toward specific demographic, geographic, or clinical characteristics. This bias can limit generalizability and reduce predictive model robustness in broader populations [3].

Training predictive models across multiple, heterogeneous cohorts has the potential to mitigate these limitations by increasing sample diversity and improving generalizability across populations [4]. Approaches such as Federated Learning (FL) offer a promising solution by enabling collaborative model training across distributed datasets without requiring data to be centrally aggregated [5]. However, FL requires a common data model (CDM) to which all local cohorts are harmonized.

Harmonizing multiple cohorts is an ongoing struggle: different cohorts, even when recording the same variables, rarely adhere to standard naming conventions, which often requires substantial effort to harmonize to a uniform standard manually. Such manual curation is time and resource-intensive and requires domain-specific experts to ensure semantic consistency and accuracy. This bottleneck not only slows down research workflows but also introduces variability depending on the expertise and interpretations of individual curators.

Numerous initiatives have been undertaken to address data harmonization, often through a CDM or standard variable schema. For instance, the Alzheimer’s Disease Data Initiative offers a standard variable system comprising 124 common variables within Alzheimer's disease (AD) cohorts. Using these variable mappings, users can harmonize cohort variables to the standard term for performing cross-cohort investigations [6]. Similarly, tranSMART is an open-source data warehouse and analytics platform that supports integration, harmonization, and analysis of translational research data using a CDM and controlled vocabularies [7]. More recent work has introduced AD-specific harmonization frameworks. AD-Mapper builds a CDM from 20 AD cohorts complemented by external CDMs and ontologies, totaling over 1200 reference variables; it leverages a BioBERT-based model to map new cohort variables and demonstrates performance improvements over simple string matching [8]. Another study harmonized ADNI and National Alzheimer’s Coordinating Center (NACC) datasets via the Alzheimer’s Disease Element Ontology (ADEO) to enable unified data element definitions and cross-cohort querying [9]. In addition, a Dutch consortium working with nine dementia cohorts applied an ETL pipeline to map local datasets into the OMOP CDM under a federated learning setup, reporting substantial benefits but also highlighting challenges with cohort-specific fields and vocabulary mismatches [10]. These approaches typically involve some degree of manual curation by the users and require an established user profile prior to harmonization. While valuable, the manual curation could potentially be further expedited through artificial intelligence (AI).

Recent advancements in language processing - particularly the rapid development and continuous improvement of large language models (LLMs) - may offer a promising solution to the challenge of labor-intensive manual curation. The application of LLM or transformer-based text embeddings for harmonization tasks has gained growing attention and has shown promising results in recent studies [[11], [12], [13], [14], [15]].

The development of new and improved LLM-based text embedding models in this field is rapid - new LLMs are published and released almost monthly. Continuous benchmarking of such models is therefore essential to ensure the best possible performance of applications that utilize them.

One of the largest and most comprehensive benchmarks to assess the performance of such embedding models is the Massive Text Embedding Benchmark (MTEB) [16]. While this benchmark includes a wide range of classification, clustering, and ranking tasks for a vast number of languages and diverse text sources, it does not cover tasks involving the automatic harmonization of clinical data. Clinical data descriptions pose a unique set of challenges due to their diverse and highly specialized terminology and vocabulary, which makes clinical data harmonization particularly complex. Accordingly, it is not clear whether LLMs performing well for various generic tasks outside the medical domain are well-suited for the harmonization of clinical data.

To fill this gap, we developed a specialized benchmark for multi-cohort variable alignment in the domain of AD, which constitutes the core contribution of this work. We evaluate five state-of-the-art text embedding models that are currently high ranking in the general MTEB embedding benchmarks, using a custom benchmark consisting of seven different AD cohorts that we map to a previously established AD CDM [8]. We discuss the challenges and limitations of automated harmonization in this domain and propose a framework of rules for clinical study metadata to guide and standardize future harmonization efforts, aiming to improve consistency, interoperability, and the quality of integrated clinical data across AD research studies.

## 2 Methods

**Fig. 1:**
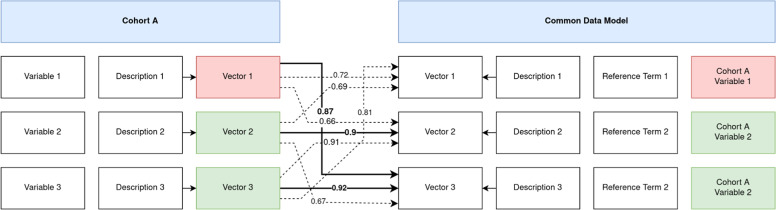
Benchmarking workflow: For each variable in each cohort, as well as each reference term in the CDM, we compute vector embeddings of the respective models. After matching them based on their cosine similarities, we compare against the respective ground truth mapping in the CDM.

To assess the feasibility of harmonizing variables across cohorts, we collected metadata (i.e., data dictionaries), consisting of variable names and variable descriptions. This was done for seven different cohort datasets in the context of AD. We evaluated five of the currently best-performing language models to benchmark their ability to automatically match cohort variable descriptions to a ground-truth description provided in a manually curated CDM based on their semantic similarity. The general approach to how matches were evaluated based on their similarity is shown in Fig. 1.

### 2.1 Cohort data

We collected cohort data from a total of seven different studies, which we will briefly describe in this section.

The Pre-symptomatic Evaluation of Experimental or Novel Treatments for Alzheimer’s Disease (PREVENT-AD) [17] cohort study is an open science dataset that collected measurements from cognitively impaired participants with a family history of AD, particularly parents or siblings. The study resulted in the collection of five years of measurements, including imaging, cerebral fluid, genetic, and clinical information [17].

The European Medical Information Framework (EMIF) [18] cohort was established to identify noninvasive biomarkers for the diagnosis of AD. Various clinical measurements, including neuropsychological tests, medication use, and comorbidities, as well as demographic variables and clinical information, were collected [18].

The GERAS cohort studies, including GERAS I, GERAS II, GERAS JAPAN, and GERAS EU, are large, prospective, multicenter observational studies designed to evaluate the clinical, social, and economic impact of AD on patients and caregivers across Europe and Japan [[19], [20], [21], [22]]. These studies collected comprehensive data on patient demographics, cognitive and functional status (e.g., Mini-Mental State Examination (MMSE)), behavioral symptoms, medication use, caregiver characteristics, healthcare resource utilization, and quality of life, with assessments conducted at baseline and multiple follow-up time points.

The PREVENT Dementia programme [23] is a multi-centre, prospective cohort study conducted across five sites in the UK and Ireland, designed to examine midlife risk factors for dementia and to identify the earliest indices of neurodegenerative disease development. The study recruited cognitively healthy participants, collecting deeply phenotyped baseline data that includes demographic information, biological samples (e.g., blood, saliva, urine, and optional cerebrospinal fluid), detailed lifestyle and psychological questionnaires, a comprehensive cognitive test battery, and multi-modal 3T MRI scans with both structural and functional sequences.

### 2.2 Common data model

To assess variable harmonization performance across various language models, we collected data dictionaries from seven different AD cohort studies. We used a previously established and publicly available AD CDM (i.e., AD-Mapper CDM), which defines 1300 core variables commonly collected in AD studies, including demographics, clinical assessments, biomarkers, and imaging data. The AD-Mapper CDM comprises the variable naming conventions of 23 distinct cohort studies that were harmonized against one another. Additionally, the CDM includes reference terms to which all variables were mapped, along with a definition for each reference term extracted from well-established ontologies, such as the Systemized Nomenclature of Medicine – Clinical Terms (SNOMED-CT) or Logical Observation Identifiers Names and Codes (LOINC) [8].

Given that evaluating automatic harmonization requires a ground-truth, we initially manually harmonized the variables from the seven cohorts to the AD CDM. Two variables were considered a match when both the variable description and value ranges were comparable. To ensure correctness, each mapping was validated by inspecting the patient-level data and corresponding value distributions. The harmonization was carried out independently by two curators, and any discrepancies were resolved through discussion until consensus was reached.

These variable mappings were then used as ground truth for evaluating the correctness of the matches suggested by different models. Since the harmonization task relied on variable descriptions provided in the metadata, we included only variables with available descriptions, as some lacked this information. Due to a low variable count for each individual GERAS study, we combined them into a single cohort study for benchmarking, which we denote as GERAS.

To evaluate the models on independent studies that may follow entirely different naming conventions, we selected two cohorts we had harmonized in our earlier work [14]. First, we identified cohorts that had been ranked as "excellent" based on a manual assessment of their metadata quality. Previously, we implemented a three-category ranking system, namely, “poor,” “adequate,” and “excellent.” The ranking was performed by two independent curators based on the clarity and comprehensiveness of the variable descriptions. For instance, a cohort data dictionary was ranked “poor” when the descriptions did not clarify what measurement had been collected or to which modality it belonged (for example, cerebrospinal fluid (CSF) vs. blood biomarker). A dictionary was ranked “adequate” when descriptions were recorded, but lacked sufficient detail to avoid ambiguity, such as “memory score” without specifying the cognitive test used. Finally, a dictionary was ranked “excellent” when the descriptions were clear, informative, and not misleading, explicitly indicating the nature of the measurement and the modality (for example, “CSF Aβ42 concentration”).

Second, we narrowed down the selection to cohorts with a comparable number of variable mappings accompanied by available variable descriptions (i.e., between 30 and 50 variables). This selection aimed to ensure comparability between cohorts and to minimize the potential bias of one cohort's performance disproportionately influencing the results. The PREVENT-AD and EMIF cohorts met these criteria and were included in our analyses. In addition, we included the PREVENT-Dementia cohort, which had not been part of our previous metadata quality ranking, but whose variable descriptions were deemed sufficiently clear and whose variable count fell within the target range.

The selected cohorts were chosen to provide a representative benchmark for evaluating variable harmonization. They encompass a range of study designs, participant populations, and data modalities, including cognitive assessments, biomarkers, imaging, and lifestyle measures. PREVENT-AD includes participants with a family history of AD, EMIF focuses on biomarker discovery across multiple clinical variables, and PREVENT-Dementia targets cognitively healthy midlife individuals. This diversity ensures that the benchmark captures heterogeneity commonly observed in AD studies and allows the assessment of harmonization approaches across different variable naming conventions and data structures. Collectively, these cohorts provide a rigorous and generalizable benchmark for evaluating harmonization performance.

An overview of the number of included variables from each cohort is shown in the supplementary material in Figure S1.

### 2.3 Large language model-based variable embeddings

We evaluated five language models, three of which ranked among the top models on the MTEB benchmark as of August 2025. We additionally evaluated OpenAI's most recent model as one of the leading proprietary competitor models in the field, as well as MiniLM as the currently most widely used lightweight open-source baseline for embedding tasks. The five evaluated models, the number of parameters, and their respective ranks in the MTEB leaderboard are shown in Table 1.

**Table 1:** Evaluated text-embedding models with corresponding MTEB leaderboard rank and model parameter size. Neither OpenAI nor Google discloses the size of their models.

Model	MTEB Leaderboard Rank	Number of Parameters
Google gemini-embedding-001 [24]	1	Undisclosed
Qwen3-Embedding-8B [25]	2 (3 + 4 for smaller variants)	8B
Linq-AI-Research/Linq-Embed-Mistral [26]	5	7B
OpenAI text-embedding-3-large [27]	16	Undisclosed
all-MiniLM-L6-v2 [28]	117	22M

For every model, we calculated vector embeddings for each cohort variable description as well as for each feature description in the CDM. Vectors were L2 normalized to unit length to ensure consistent scaling across different models. The normalized vectors were then matched based on their cosine similarity to each possible CDM vector (see Fig. 1).

We measured model performances based on:

a) Accuracy of Zero-Shot classification, defined as the proportion of variables correctly matched to their corresponding feature in the CDM based on their highest cosine similarity (Table 2).

**Table 2:** AUPRC, zero-shot accuracy, and weighted composite scores for each model across cohorts. Zero-shot accuracy measures correct CDM matches without prior training. The composite score combines metrics weighted by cohort size. Cohorts in the table are ordered based on the rank in the MTEB.

Model	**Cohort**	**AUPRC**	**Zero-Shot**	**Composite Score**
Google: gemini-embedding-001	GERAS	**0.43**	0.62	0.40
	PREVENT Dementia	0.29	0.48	
	PREVENT-AD	0.28	0.28	
	EMIF	0.21	0.42	
**Qwen:** **Qwen3-Embedding-8B**	GERAS	0.29	0.46	0.30
	PREVENT Dementia	0.29	0.36	
	PREVENT-AD	0.21	0.22	
	EMIF	0.11	0.35	
**Linq-AI-Research: Linq-Embed-Mistral**	GERAS	0.39	0.35	0.37
	PREVENT Dementia	**0.42**	0.36	
	PREVENT-AD	**0.29**	0.25	
	EMIF	**0.35**	0.54	
**OpenAI: text-embedding-3-large**	GERAS	0.35	**0.66**	**0.43**
	PREVENT Dementia	0.31	**0.52**	
	PREVENT-AD	0.28	**0.39**	
	EMIF	0.32	0.52	
**UKP Lab:** **all-MiniLM-L6-v2**	GERAS	0.35	0.56	0.40
	PREVENT Dementia	0.31	0.44	
	PREVENT-AD	**0.29**	0.34	
	EMIF	0.3	**0.58**
b) Area Under Precision Recall Curve (AUPRC) for all variable mappings depending on the similarity range of each mapping (Fig. 2, Table 2).

**Fig. 2:**
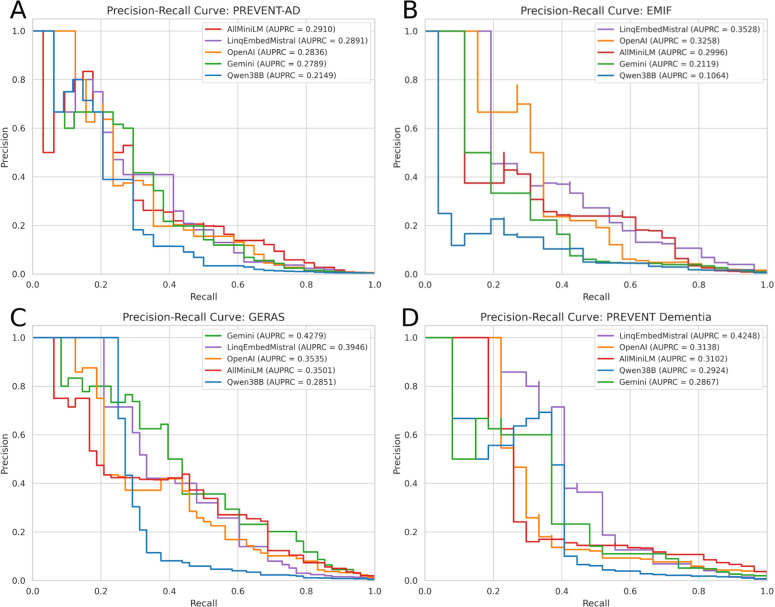
Precision-Recall curves and computed AUPRC for all models per cohort. Evaluated cohorts are PREVENT-AD (A), EMIF (B), a combination of GERAS studies (C), and PREVENT Dementia (D).

*Precision* and *Recall* were calculated for 100 thresholds of vector cosine similarities from 1.0 to 0.01. This approach yielded 100 data points for each precision and recall. Included variables that correctly matched any one-to-one or upper-level concept in the CDM were considered as *True Positives (TP)*. Although the automatic mapping procedure was performed on a one-to-one basis, the manual curation occasionally harmonized multiple cohort variables to the same reference concept. For example, for a cohort where the apolipoprotein E (APOE) genotype was recorded separately, two cohort variables were mapped from that cohort to the reference term “APOE” in the CDM. In this case, if either of those variables were mapped to the “APOE” variable, we counted that variable as a correct mapping. Variables included in the threshold that did not correctly match any CDM variable were considered *False Positives (FP)*; variables excluded due to the respective threshold that had a potential match in the CDM were considered *False Negatives (FN)*. We excluded any variables that did not have any potential match in the CDM prior to vector computation.

We computed precision and recall for each threshold using the standard formulas:

$$Precision\;=\;\frac{TP}{TP+FP}Recall\;=\;\frac{Tp}{TP+FN}$$

While a high minimum similarity threshold (i.e., only consider vectors of “perfect” similarity of 1.0) will likely result in high precision and low recall, the opposite will likely lead to high recall but lower precision. When plotting these two measures against each other, the AUPRC can be used to determine how well a model is able to match similar descriptions other than the best match, since the correct match may not always have the highest similarity score, but could still be among the most similar candidates.

To reach a statistically meaningful assessment of language model capabilities, results included a total of eight scores per model, with the two metrics introduced above for each of the four evaluated cohorts. To enable a comprehensive comparison across all metrics and cohorts, we additionally computed a weighted composite score as follows:

$$score=\;\sum_{cohort}\left(0.5\;\cdot \;AUPRC_{cohort}+\;0.5\;\cdot \;ZeroShotAccuracy_{cohort}\right)\;\cdot \;\frac{\#Vars_{cohort}}{\#Vars_{total}}$$

We assigned equal weights to AUPRC and zero-shot accuracy because they capture complementary but equally important aspects of model performance: AUPRC reflects ranking quality across different thresholds, while zero-shot accuracy measures direct classification success based on the highest similarities without threshold tuning. Giving them equal contribution ensures that the composite score balances both threshold-independent ranking and practical accuracy. Cohort-level weighting by variable count was applied to ensure that cohorts contributing more data have a proportionally larger influence on the composite score, reflecting the practical impact of model performance on the overall harmonization task. This approach prevents smaller cohorts with few variables from disproportionately affecting the composite score, which could misrepresent overall performance. The final composite score ranged from 0 to 1, and we report this score for all models in Table 2, alongside the individual AUPRC and zero-shot accuracy values.

OpenAI’s *text-embedding-3-large* model produced slightly different embedding vectors across runs when retrieved via the Python API, due to floating-point precision differences. This occasionally led to flips in similarity rankings for individual comparisons. To mitigate this, we averaged the results over 10 runs. These variations affected only isolated mappings and did not influence the model’s overall aggregated performance score.

## 3 Results

We first evaluated the total performance of each individual benchmarked model based on its combined performance across both metrics (zero-shot accuracy, AUPRC) over all cohorts using the composite score introduced in the previous section. In terms of their total score, OpenAI’s *text-embedding-large* performed best with a total score of 0.43, followed closely by Google's *gemini-embedding-001* model and all-MiniLM tied with a score of 0.40 (Table 2).

Out of the open-source models, *all-MiniLM-L6-v2* performed best with a composite score of 0.40. *Linq-Embed-Mistral* followed closely with a composite score of 0.37, with only the *Qwen3-Embedding-8B* model falling short with an overall score of 0.30.

In terms of zero-shot classification performance, OpenAI’s *text-embedding-large* model outperformed all other evaluated models for all evaluated cohorts except for EMIF, with a total zero-shot accuracy of 0.66 for the combined GERAS cohorts, an accuracy of 0.52 for PREVENT Dementia, and 0.39 for PREVENT-AD. For the EMIF cohort, the all-MiniLM-L6-v2 model performed best with a zero-shot accuracy of 0.58, closely followed by the Linq-Embed-Mistral model with an accuracy of 0.54.

Google's *gemini-embedding-001* model performed slightly worse than OpenAI’s model for the first two cohorts (both −0.04). Though being a significantly smaller model (less than 1/300 of parameters) than the other two tested open-source models, as well as ranking the lowest (rank 117) on the MTEB, the all-MiniLM model outperformed both the LinqEmbedMistral and Qwen3 models for all cohorts in terms of zero-shot classification accuracy and even the leading OpenAI model specifically for the EMIF cohort.

**Fig. 3:**
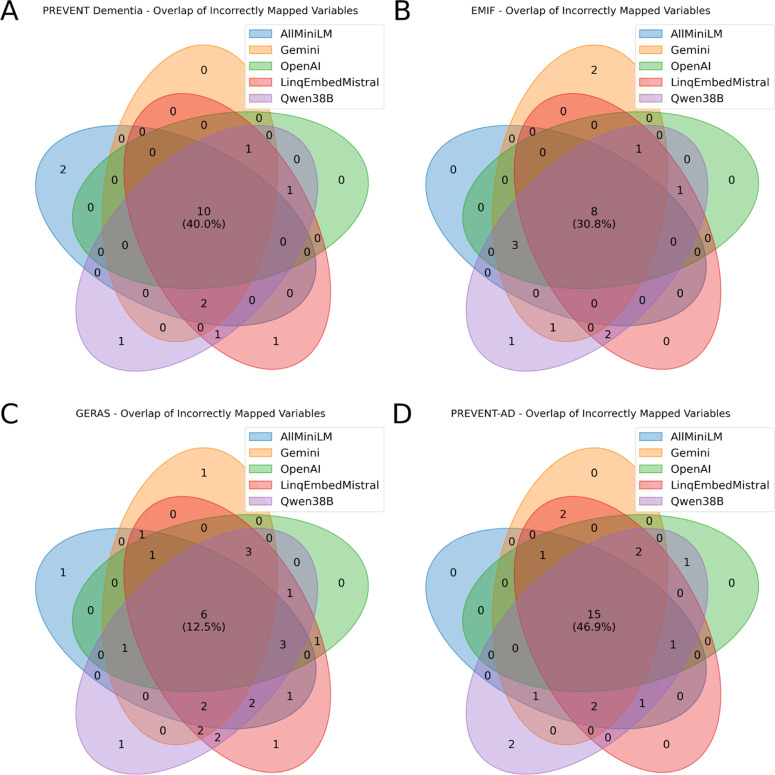
Overlap of incorrectly mapped variables (zero-shot) across models for each cohort. Panels A–D correspond to the PREVENT Dementia, EMIF, GERAS, and PREVENT-AD cohorts, respectively. The Venn diagrams show the number of shared and unique mapping errors among the five models (AllMiniLM, Gemini, OpenAI, LinqEmbedMistral, Qwen38B).

While there were significant differences in terms of zero-shot accuracy for the individual models, there was also a high overlap in cohort variable descriptions that could not be correctly mapped by any of the evaluated models. We show a visualisation of incorrect variable overlaps for each evaluated model for each cohort in Fig. 3. For the GERAS studies, a total of 6 variables could not be correctly mapped by any model based on the most similar description vector, which corresponds to a fraction of 12.5 % of all recorded variables. Other cohorts show a bigger relative and absolute number of variables that were incorrectly matched based on the zero-shot approach. EMIF had a total of 8 or 30.8 % of variables incorrectly matched based on the most similar embedding for all models, and PREVENT Dementia had a total of 10 variables corresponding to 40 % of all variables. PREVENT-AD showed the highest misclassification rate, with 15 variables (46.9 %) mapped incorrectly by all models.

A closer inspection of these errors reveals common patterns. For example, variables related to age and time points, such as “Age when assessed” (EMIF), “Age in months at the time of test” (PREVENT-AD), or “Age of participant” (PREVENT Dementia), were frequently mismatched due to subtle differences in phrasing. Long or complex variable descriptions, such as “Long-form variable for disease code for any selected diagnoses…” (GERAS) or “Has the participant shown a low cognitive performance…determined by clinical consensus” (PREVENT-AD), also led to misclassifications by the models. In addition, modality-specific biomarkers like “Neurofilament light values of central CSF analyses” (EMIF) or “Beta-amyloid 1–42 concentration in CSF” (PREVENT-AD) were often mismatched, reflecting difficulties in distinguishing between similar biological measurements across different sample types. These examples illustrate that systematic mismatches often arise for variables with subtle wording differences, unusually detailed descriptions, or modality-specific context, highlighting the limitations of current text embedding models in capturing nuanced clinical semantics. We propose technical guidelines on data descriptions based on commonly mismatched variables in the Discussion section.

The AUPRC (Fig. 2) showed mixed results depending on both the specific models and cohorts; Google’s *gemini-embedding-001* performed the best for the GERAS cohorts with an AUPRC of 0.43. For the other 3 cohorts, the Linq-Embed-Mistral model showed the highest AUPRC of 0.42 for PREVENT Dementia, 0.35 for EMIF, and 0.29 for PREVENT-AD, tied with the all-MiniLM model.

As new text-embedding models with improved capabilities are emerging, with increased frequency, in the public domain, choosing the right model, especially for time and resource-intensive tasks such as data harmonization, can be challenging. To facilitate the benchmarking of new models, we provide a Python library named "Alzheimer's Disease Harmonization Text Embedding Benchmark” (ADHTEB) [29] to enable benchmarking of future models. We also publish an interactive leaderboard^2^ showcasing top-performing models. Users can benchmark their own custom models against this novel benchmark with minimal effort, whilst retaining the option to publish their results to the leaderboard with a single line of code.

## 4 Discussion

Based on the results of the previous section, the resulting ranking of our benchmarks differs substantially from the general benchmark results shown in the MTEB. While we also found Gemini's embedding model performing consistently well across all tasks, it was outperformed by OpenAI’s model for all cohorts for the zero-shot classification task, as well as two of four cohorts based on the overall AUPRC.

Two of the recently published, high-ranked open-source models performed substantially worse than in the MTEB rankings. Notably, the Qwen3 model that held the second to fourth spot in the MTEB ranking performed worst in terms of the computed overall score. The widely used, but arguably older model, all-MiniLM, interestingly performed best out of the tested open-source models, despite its small parameter size. This could be potentially explained by either:

a) **Overfitting to MTEB:** Some models may have been fine-tuned to perform well specifically on MTEB tasks, which could limit their generalization to other applications, such as the harmonization task simulated in our benchmark.
b) **Task-specific differences:** Our harmonization benchmark likely differs fundamentally from the tasks included in MTEB, both in terms of domain and structure, which may favor different model capabilities.

In either case, these findings strongly support the relevance and potential value of our proposed benchmark. Whether the performance gap arises from MTEB-specific fine-tuning or from fundamental differences in task requirements, it highlights that general-purpose benchmarks may not adequately capture performance in real-world, domain-specific applications such as data harmonization. These observations also highlight the potential benefits of developing specialized or fine-tuned AI models for dementia-related data, as further discussed in Section 4.1.

The results obtained from different models and evaluated across distinct cohorts indicate that harmonization accuracy is strongly influenced by the quality of variable descriptions. In some instances, variables were mapped to incorrect modalities. For example, the variable “Neurofilament light values of central CSF analyses” was incorrectly harmonized to “Neurofilament Light Chain (NfL) in Plasma” in the EMIF cohort by all evaluated models. Moreover, in many cases where harmonization failed, the absence of shared terminology across variable descriptions was a contributing factor.

A key factor for mismatches in automated mappings is the general lack of variable naming conventions in clinical metadata. Establishing and adhering to consistent naming conventions when defining cohort metadata and variable descriptions can not only facilitate manual harmonization but also enhance the performance of embedding-based approaches for automated data harmonization. Based on our observations obtained in this benchmark study, we propose the following general recommendations to improve metadata quality and semantic clarity:

- Metadata should be provided in a machine-readable format (e.g., JSON, CSV with standardized headers) to facilitate automated parsing and harmonization. Providing structured metadata allows both human users and computational tools to access and process variable information consistently.
- A common character encoding (e.g., UTF-8) should be used for all metadata to ensure consistent interpretation across systems. This helps avoid issues with special characters, symbols, or accented letters that may otherwise cause parsing errors or mismatches during harmonization.
- Descriptions should always specify the modality from which the variable originates (e.g., CSF). Otherwise, for variables that can be measured across different modalities, such as Aβ, harmonization may inadvertently group distinct measurements together.
- Variables intended to be mapped to higher- or lower-level concepts (e.g., hippocampus volume vs. left hippocampus volume) should be described in sufficient detail to prevent higher-level measurements from being mistakenly mapped to more specific ones.
- Unnecessary wording can lead to misleading matches. For example, the variable “Age in months at the time of test” was incorrectly mapped to “The month in which a person was born.” Since measurement units are often recorded in a separate column, excluding them from the variable description may improve mapping accuracy and help avoid such errors.

Based on the zero-shot accuracies of the evaluated cohort to CDM mapping, even though text-embedding models may facilitate the task of data harmonization, it is apparent that they cannot fully replace human curation. Although models may not always match the correct variables based on the description with the closest cosine similarity, the correct match is, in most cases, still among the most similar variable matches. Harmonization workflows that utilize text-embedding similarities can thus be better applied to enable *semi-automatic* harmonization, where a human in the loop may choose from a list of variables that are high-ranking in terms of their semantic similarity. By narrowing down potential correct matches using a ranked list of promising terms, the overall curation effort is significantly expedited by reducing the search space and guiding human experts toward the most semantically relevant candidates. For example, when harmonizing a cohort with 100 variables, instead of reviewing all possible matches individually, the user could focus on the top 10 most semantically similar candidates, greatly reducing effort while maintaining accuracy.

The computed AUPRC provides a way to assess how well variables are ranked, in our implementation, based on their absolute cosine similarities. Although we can see trends in the results that match those of the zero-shot classification performance, the results for the different models vary between cohorts. A possible explanation is the different wording for different cohorts; some cohorts may follow different naming conventions that favor different models trained on different sets of data. Another potential reason for the performance discrepancy in terms of AUPRC when compared to zero-shot accuracies, particularly for the PREVENT Dementia and EMIF cohorts, could be a low number of variables. To address this, future studies will include additional cohorts, which should also help reduce variance caused by differences in variable descriptions across cohorts.

### 4.1 Limitations and future work

Our benchmark exclusively contains cohorts in the domain of AD, which may in itself be biased toward certain naming conventions or variable formulations. Hence, we now plan to explore extending it with cohorts in other related neurodegenerative diseases. A natural next step would be to include Parkinson’s disease, as our previous work has shown that there is a substantial overlap in variables while introducing new domain-specific elements [14].

In our current approach, we evaluate the capabilities of LLMs with regard to text embeddings. A possible extension could involve leveraging LLMs not only for embedding generation but also for actively selecting the most appropriate matches among candidate variables. Instead of relying solely on cosine similarity to determine the best match, a more advanced harmonization pipeline could employ the full reasoning capabilities of LLMs in a second stage. This two-stage approach would enable the model to go beyond surface-level similarity and incorporate domain-specific logic and latent cues present in variable descriptions, leading to more robust and interpretable harmonization decisions. LLMs could also be further utilized to interface between data curators and harmonization outputs by generating natural language explanations for mapping decisions or giving feedback on inconsistent manual mappings. Additionally, when individual AI models perform sub-optimally, researchers could leverage complementary strategies, such as ensemble approaches that combine outputs from multiple models or cross-validation with manually curated reference mappings. Incorporating human-in-the-loop curation at critical decision points can further improve reliability. Moreover, model performance could be enhanced by iterative fine-tuning using domain-specific examples or by integrating structured ontologies as additional guidance. These strategies collectively allow AI to support harmonization more effectively, even when single-model performance is limited.

## 5 Conclusion

While AI and especially LLM-assisted systems can potentially facilitate and accelerate the manual curation effort, it is important to promote transparency both in the model selection itself as well as in the choices made by the model during harmonization. Our benchmark contributes to this goal by systematically evaluating model behavior in a domain-specific setting, thereby offering insight into each model’s strengths, limitations, and suitability for real-world harmonization tasks. Nonetheless, a human expert should always remain in the loop to review and validate model outputs, ensuring that final decisions are made by curators rather than delegated entirely to automated systems.

In alignment with these considerations, our work introduces a benchmark that operationalizes transparency in the context of variable harmonization. A domain-specific benchmark, as presented, is essential to address the unique challenges posed by harmonization workflows of general-purpose benchmarks, such as the MTEB, in AD. We outline key limitations of automated data harmonization and propose best practices for naming conventions to be able to still leverage embedding-based harmonization workflows. Finally, we present our benchmark approach as an accessible, open-source Python package that can be used to evaluate new and upcoming models in the future.

## Glossary

**AD** (Alzheimer's Disease): A neurodegenerative disease characterized by rapid cognitive decline.

**ADEO** (Alzheimer’s Disease Element Ontology): Standard vocabulary for Alzheimer’s disease research data.

**ADNI** (Alzheimer's Disease Neuroimaging Initiative): A large, multicenter study collecting clinical, imaging, genetic, and biomarker data to investigate Alzheimer’s disease progression and improve early diagnosis.

**AI** (Artificial Intelligence): An application or software able to perform tasks or produce output that would usually require some degree of human intelligence.

**AUPRC** (Area Under Precision Recall Curve): A metric that measures model performance based on precision and recall across different thresholds.

**CDM** (Common Data Model): A standardized framework for structuring and describing data elements to enable interoperability across datasets.

**CSF** (Cerebrospinal Fluid): Clear fluid of the brain and spinal cord.

**CSV** (Comma Separated Values): A common file format for tabular data storage.

**EMIF** (European Medical Information Framework): European cohort combining clinical and biomarker data to study Alzheimer’s disease progression and risk factors.

**ETL** (Extract, Transform, Load): A common data processing practice, consisting of data extraction from multiple sources, transformation into a standard format, and storage into a database or data repository.

**FL** (Federated Learning): An application of several decentralized machine learning models that is trained to produce one centralized prediction or output.

**GERAS**: Observational studies of Alzheimer's disease and dementia.

**JSON** (JavaScript Object Notation): A text-based data transfer format, commonly used to transfer data between applications.

**LLM** (Large Language Model): A transformer-based Neural Network with a high amount of parameters, trained on a large corpus of texts to understand human language.

**LOINC** (Logical Observation Identifiers Names and Codes): Standard system for identifying and coding laboratory tests and clinical measurements.

**MMSE** (Mini-Mental State Examination): A test assessing cognitive function and screening for cognitive impairment.

**MTEB** (Massive Text Embedding Benchmark): A generalized, public, and well-established benchmark of different text embedding models.

**NACC** (National Alzheimer’s Coordinating Center): A consortium collecting and sharing standardized clinical and neuropathological data from Alzheimer’s disease research centers.

**OMOP** (Observational Medical Outcomes Partnership): A standardized Common Data Model used primarily in health care and patient-related data processing.

**PREVENT-AD** (Pre-symptomatic Evaluation of Experimental or Novel Treatments for Alzheimer’s Disease): A longitudinal study focused on identifying early biomarkers and testing preventive interventions in individuals at risk for Alzheimer’s disease.

**SNOMED CT** (Systematized Nomenclature of Medicine – Clinical Terms)**:** An international standardized terminology for indexing of medical terms.

## Funding sources

SYNTHIA: Synthetic Data Generation framework for integrated validation of use cases and Al healthcare applications.

This project is supported by the Innovative Health Initiative Joint Undertaking (IHI JU) under grant agreement No 101172872. The JU receives support from the European Union's Horizon Europe research and innovation programme, COCIR, EFPIA, Europa Bío, MedTech Europe, Vaccines Europe and DNV. The UK consortium partner, The National Institute for Health and Care Excellence (NICE) is supported by UKRI Grant 10132181.

## Disclaimer

Funded by the European Union, the private members, and those contributing partners of the IHI JU. Views and opinions expressed are however those of the author(s) only and do not necessarily reflect those of the aforementioned parties. Neither of the aforementioned parties can be held responsible for them.

## Declaration of generative AI and AI-assisted technologies in the writing process

During the preparation of this work, the author(s) used OpenAI's ChatGPT in order to proofread and stylistically refine the text. The tool was used solely for spelling, grammar correction, and rephrasing assistance. All scientific content, data interpretation, and conclusions were generated independently by the authors without AI involvement. After using this tool/service, the author(s) reviewed and edited the content as needed and take(s) full responsibility for the content of the publication.

## CRediT authorship contribution statement

**Tim Adams:** Conceptualization, Formal analysis, Investigation, Methodology, Software, Software, Validation, Visualization, Writing – original draft. **Yasamin Salimi:** Writing – original draft, Visualization, Resources, Methodology, Investigation, Formal analysis, Data curation. **Mehmet Can Ay:** Writing – review & editing, Visualization, Software, Investigation, Formal analysis, Data curation. **Diego Valderrama:** Writing – review & editing, Resources, Data curation. **Marc Jacobs:** Writing – review & editing, Conceptualization. **Holger Fröhlich:** Conceptualization, Funding acquisition, Investigation, Methodology, Project administration, Supervision, Writing – review & editing.

## Declaration of interests

The authors declare the following financial interests/personal relationships which may be considered as potential competing interests:

Holger Froehlich reports that financial support was provided by Gates Ventures. Tim Adams, Yasamin Salimi, and Diego Valderrama report that administrative support was provided by Gates Venture. If there are other authors, they declare that they have no known competing financial interests or personal relationships that could have appeared to influence the work reported in this paper.

## Appendix Supplementary materials

Image, application 1

## Footnotes

2 https://adhteb.scai.fraunhofer.de/
