# Supplementary material for: A benchmark of text embedding models for semantic harmonization of Alzheimer's disease cohorts
Source: J Prev Alzheimers Dis. 2025 Dec 1;13(1):100420. doi: 10.1016/j.tjpad.2025.100420 (PMC12811766; doi:10.1016/j.tjpad.2025.100420)
Supplement: Supplementary file 1 [file mmc1.pdf]

Supplementary Material

A Benchmark of Text Embedding Models for Semantic Harmonization of Alzheimer's Disease Cohorts

Tim Adams[a,‡], Yasamin Salimi[a,‡], Mehmet Can Ay[a], Diego Valderrama[a], Marc Jacobs[a], Holger Fröhlich[a,b,*]

‡ **equal contribution**
* **corresponding author:** Holger Fröhlich <holger.froehlich@scai.fraunhofer.de>, Schloß Birlinghoven, Sankt Augustin 53757, Germany

[a] Department of Bioinformatics, Fraunhofer Institute for Algorithms and Scientific Computing (SCAI), Sankt Augustin 53757, Germany
[b] Bonn-Aachen International Center for IT, Rheinische Friedrich-Wilhelms-Universität Bonn, Bonn, Germany

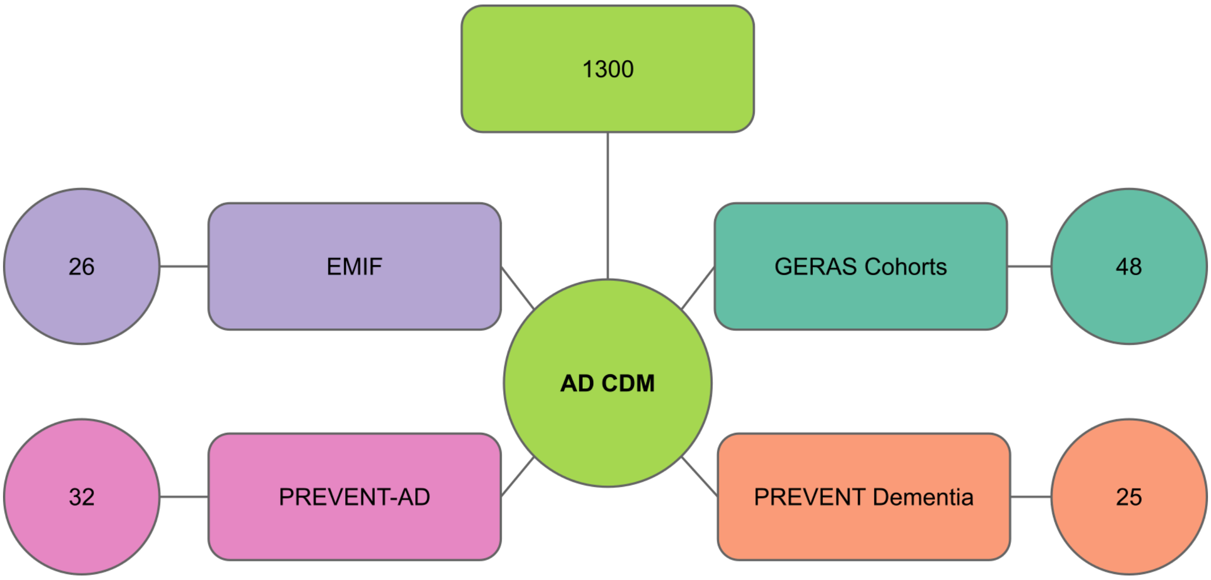

Figure S1: The number of mapped variables from each cohort study to the AD CDM.
